# Differences between leukemic arthritis and juvenile idiopathic arthritis

**DOI:** 10.1186/s12969-023-00836-5

**Published:** 2023-05-31

**Authors:** Alfonso Ragnar Torres Jimenez, Eunice Solis Vallejo, Adriana Ivonne Cespedes Cruz, Julia Veronica Ramirez Miramontes, Guadalupe del Consuelo Cortina Olvera, Alejandra Velazquez Cruz, Berenice Sanchez Jara

**Affiliations:** 1 Department of Pediatric Rheumatology, National Medical Center, La Raza, IMSS, Vallejo Y Jacarandas, Colonia La Raza, Azcapotzalco, Mexico City, 02990 México; 2 Department of Pediatric Hematology, National Medical Center La Raza, IMSS, Mexico City, México

**Keywords:** Leukemic arthritis; juvenile idiopathic arthritis, Neutropenia, Acute lymphoblastic leukemia, Arthritis

## Abstract

**Objectives:**

To determine the clinical and laboratory differences between leukemic arthritis (LA) and juvenile idiopathic arthritis (JIA) at the onset of the disease.

**Material and methods:**

Patients under 16 years of age, both genders, who presented for the first time to the pediatric rheumatology service with a diagnosis of probable JIA, with arthritis and without peripheral blood blasts, in which the final diagnosis was acute lymphoblastic leukemia (ALL) or JIA. The clinical and laboratory characteristics of the patients were compared, chi-square and relative risk were used for categorical variables, and the Mann–Whitney U and T-test for the comparison of means between groups. A binary logistic regression model was developed to differentiate leukemic arthritis from JIA.

**Results:**

A total of 76 patients, 14 with LA and 62 with JIA, were analyzed. The mean age at diagnosis was lower in the leukemic arthritis group, the female gender prevailed in the JIA group, and the time to onset of symptoms was lower in the leukemic arthritis group. Patients with leukemic arthritis showed increased pain intensity, fever, weight loss, nocturnal diaphoresis, lymph node enlargement, hepatosplenomegaly, and pain that did not improve with analgesic administration. Laboratory parameters with statistical significance were the presence of anemia, leukopenia, and neutropenia. The platelet count was significant but in a low normal value, compared to the JIA. A binary logistic regression model was developed to differentiate leukemic arthritis from JIA. The probability associated with the statistic (Chi-square) was 0.000, and the Cox and Snell R2 and Nagelkerke R2 values were 0.615 and 1, respectively. The developed model correctly classified 100% of the cases.

**Conclusions:**

The diagnosis of acute lymphoblastic leukemia should be ruled out in patients who present with arthritis and hematological alterations, mainly leukopenia and neutropenia, with joint pain disproportionate to the degree of arthritis, predominantly at night and that does not improve with the use of analgesics, fever, lymph nodes, and hepatosplenomegaly. Criteria are suggested to differentiate both diseases.

## Introduction

Acute lymphoblastic leukemia (ALL) is the most common type of cancer in children, and juvenile idiopathic arthritis (JIA) is the most common chronic rheumatic disease in childhood. Most patients with leukemia present with symptoms of bone marrow failure (anemia, leukopenia, thrombocytopenia), however; musculoskeletal manifestations occur in approximately 30% of cases, manifesting as bone pain, arthralgia, myalgia, limp or arthritis. Sometimes these joint symptoms are the only ones present, leading to a misdiagnosis of juvenile idiopathic arthritis, with the consequent use of steroids or disease-modifying drugs, which increase morbidity and mortality. We present a study that shows the clinical and laboratory differences between these 2 diseases, and we propose criteria to help the clinician to easily differentiate between them [1–4].

## Material and methods

Patients under 16 years of age, who initially presented to the pediatric rheumatology service of the General Hospital of the La Raza National Medical Center, from January 2013 to December 2022, with arthritis, who did not show blasts in peripheral blood, in whom the final diagnosis was juvenile idiopathic arthritis or acute lymphoblastic leukemia. Arthritis is defined as the presence of joint inflammation and effusion. The clinical manifestations evaluated were: age, gender, time of onset of symptoms, pain intensity, presence of nocturnal pain, remission with analgesics, fever, weight loss, nocturnal diaphoresis, lymph node enlargement, and hepatosplenomegaly. The laboratory variables evaluated were: hemoglobin, leukocytes, neutrophils, lymphocytes, platelets, erythrocyte sedimentation rate and C-reactive protein. The clinical and laboratory variables of both groups were compared. Leukemias were classified according to the French-American-British classification of acute lymphoblastic leukemia** (**FAB) classification, and juvenile idiopathic arthritis according to the International League of Associations for Rheumatology (ILAR) criteria [5, 6].

The SPSS 25 statistical software was used. The data were expressed in percentages for each area of analysis; chi-square and relative risk for qualitative variables, Mann–Whitney U test and T-test for the comparison of means between the groups. It was considered significant with a *p* < 0.05. A model was developed using binary logistic regression; the enter mode was used. The model's goodness of fit was verified using the Hosmer and Lemeshow's test, Cox and Snell's R2 and Nagelkerke's R2 were calculated.

## Results

Data from a total of 76 patients were analyzed, 14 with leukemic arthritis and 62 with JIA (Table 1). The mean age in the leukemic arthritis (LA) group was 8.1 years (3–14 years) and in the JIA group, 10.3 years (2–15 years) (*p* = 0.084), the female gender predominated in the JIA group (*p* = 0.026). The pattern of arthritis in the group of patients with leukemia was polyarticular in 43% and oligoarticular in 50% (migratory in 71% of these). In the JIA group, the polyarticular pattern was the most frequent (85%) (Table 2).

**Table 1:** Subtypes of leukemia and JIA

FAB	*n* = 14(%)	ILAR	*n* = 62 (%)
**ALL L1**	12 (86%)	**Oligoarticular**	**4 (6%)**
**ALL L2**	2 (14%)	**Polyarticular RF –**	**27 (44%)**
**ALL L3**	0	**Polyarticular RF + **	**24 (39%)**
		**Enthesitis related arthritis**	**3 (5%)**
		**Systemic arthritis**	**4 (6%)**

**Table 2:** Pattern of joint affection

	**Leukemic arthritis**	**JIA**
Oligoarticular	50% (migratory in 5)	10%
Polyarticular	43%	85%
Enthesitis related arthritis	7%	5%

Oligoarthritis: 1 to 4 joints, Polyarthritis: 5 or more joints, Migratory arthritis: swelling in one or two joints, with resolution over a few days. As the symptoms resolve, similar symptoms emerge in another joint, usually in an asymmetric location

Comparing the leukemic arthritis group with the JIA group, we found that the onset time of symptoms was 4.2 months vs. 9.1 months (*p* = 0.017) and the pain intensity was 10 vs. 7 on the pain analogue scale (*p* = 0.0001), the presence of nocturnal pain that awakens the patient 79% vs 10% (*p* = 0.0001, RR 12.7), pain remission with analgesics 0% vs 74% (*p* = 0.0001, RR 1.8), fever 64% vs 6% (*p* = 0.0001, RR 8.7), weight loss 86% vs 19% (*p* = 0001, RR 13), nocturnal sweating 50% vs 0% (*p* = 0.0001, RR 9.8), lymph nodes 86% vs 3% (*p* = 0.0001, RR 26), hepato-splenomegaly 57% vs 3% (*p* = 0.0001, RR 8.8) respectively. Anemia was observed in 42% vs 8% (*p* = 0.001, RR 4.4), leukopenia in 71% vs 2% (*p* = 0.0001, RR 14.7), neutropenia 92% vs 0% n (*p* = 0.0001, RR 63), thrombocytopenia in 43% vs 0% (*p* = 0.0001, RR 8.7), mean hemoglobin value was 11.5 g/dL vs 13 g/dL (*p* = 0.009), leukocytes 3508 mm3 vs 9111 mm3 (*p* = 0.0001), neutrophils 695 mm3 vs 5143 mm3 (*p* = 0.0001), platelets 191,000 mm3 vs 378,000 mm3 (*p* = 0.0001) in leukemic arthritis and JIA group respectively. No difference was found in the values of lymphocytes, erythrocyte sedimentation rate and C-reactive protein (Table 3).

**Table 3:** Clinical and laboratory characteristics in patients with leukemic arthritis vs JIA

Variable	Leukemic arthritis (*n* = 14)	JIA (*N* = 62)	*p value*	RR (IC 95%)
Age (years)	8.1 (3–14)	10.3 (2–15)	0.084	
Female gender	50%	79%	*0.026	
Time of symptoms onset (months)	4.29 (1–12)	9.15 (1–72)	*0.017	
Pain intensitiy VAS (0–10)	10	7 (2–10)	*0.0001	
Night time pain	79%	10%	*0.0001	12.7 (4–40)
Pain relief with analgesics	0%	74%	*0.0001	0.53(0.38–0.74)
Fever	64%	6.5%	*0.0001	8.7 (3.4–21.7)
Nocturnal diaphoresis	50%	0%	*0.0001	9.8 (4.8–19.8)
Weightloss	86%	19%	*0.0001	13 (3.1–53.6)
Lymphadenopathies	86%	3%	*0.0001	26 (6.6–105)
Hepatosplenomegaly	57%	3%	*0.0001	8.8 (3.8–20)
Rheumatoid factor	0%	25%	0.011	
Anemia (hb < 11 g/dL)	42%	8%	*0.001	4.4 (1.9–10.3)
Hemoglobin g/dL	11.5 (8.3–14.7)	13 (8.4–16.4)	0.009	
Leukopenia (< 4000 mm^3^)	71%	2%	*0.0001	14.7 (5.6–38)
Leukocytes mm^3^	3508 (1610–5560)	9111 (4450–24,630)	*0.0001	
Neutropenia (< 1500 mm^3^)	92%	0%	*0.0001	63 (9–440)
Neutrophils mm^3^	695 (0–1820)	5413 (2070–22,130)	*0.0001	
Lymphocytes mm^3^	2488 (460–4230)	2787 (1320–9280)	0.583	
Thrombocytopenia (< 150 × 10^3^/mm^3^)	43%	0%	*0.0001	8.7 (4.5–16.7)
Platelets × 10^3^/mm^3^	191 (53–406)	378 (196–759)	*0.0001	
ESR mm/hr	34 (20–67)	27.7 (2–66)	0.302	
CRP	39 (5–100)	25 (0–226)	0.247	

*ESR* Erythrocyte sedimentation rate, *CRP* C reactive protein, *VAS* Visual Analogue Scale

^*^Statistically significant

A binary logistic regression model was developed to differentiate leukemic arthritis from JIA. The probability associated with the statistic (Chi-square) was 0.000, and the Cox and Snell R2 and Nagelkerke R2 values were 0.615 and 1, respectively. The developed model correctly classified 100% of the cases (Tables 4 and 5).

**Table 4:** Sensitivity and specificity of clinical and laboratory criteria

**Variables**	Sensitivity	Specificity	Correctly classified
2 or more clinical variables	84%	100%	86.8%
3 or more clinical variables	93%	100%	94.7%
1 or more laboratory criteria	90%	100%	92.1%
1 or more laboratory criteria without anemia	98%	100%	98.7%
1 or more clinical variables 1 or more laboratory criteria	92%	100%	93.4%
2 or more clinical variables 1 or more laboratory criteria	95%	100%	96.1%
3 or more clinical variables 1 or more laboratory criteria	97%	100%	97.4%
1 or more clinical variables 1 or more laboratory criteria without anemia	98%	100%	98.7%
2 or more clinical variables 1 or more laboratory criteria without anemia	100%	100%	100%
3 or more clinical variables 1 or more laboratory criteria without anemia	100%	100%	100%

**Table 5:** Proposed criteria for the diagnosis of leukemic arthritis

In a patient with arthritis, consider the diagnosis of leukemic arthritis and perform bone marrow aspirate if at least 1 laboratory and 2 clinical criteria are present
**Laboratory criteria**
• Leukopenia < 4000/µL
• Thrombocytopenia < 150,000/µL
• Neutropenia < 1500/µL
**Clinical criteria**
• Pain intensity 10/10
• Night pain
• Pain that does not relieve with analgesics
• Fever
• Weightloss
• Nocturnal diaphoresis
• Lymphadenopathy
• Hepatosplenomegaly

Chi square *p* = 0.000, Hosmer and Lemeshow *p* = 1.00, Cox and Snell R^2^ = 0.615, Nagelkerke R^2^ = 1.000, Sensitivity 100% Specificity 100%

## Discussion

Musculoskeletal pain is a common symptom, affecting 10–20% of school-age children. Most of these complaints are benign in nature. However, some hematological diseases have been associated with rheumatic symptoms. These diseases can be benign (sickle cell disease, thalassemia, hemochromatosis, hemophilic arthropathy) or malignant (leukemia, lymphoma, multiple myeloma, neuroblastoma) [1].

Leukemia is the most common childhood cancer (30–40% of cases) and acute lymphoblastic leukemia is the most common subtype (80%), with a peak incidence between 2–6 years of age. Most patients with leukemia present with pallor, fever, lymphadenopathy, visceromegaly, and a variety of hematologic abnormalities (anemia, thrombocytopenia, leukopenia, leukocytosis, and peripheral blood blasts). However, one-third of pediatric patients with acute lymphoblastic leukemia initially present with symptoms associated with the musculoskeletal system [2]. Localized or diffuse joint pain, claudication, monoarthritis or polyarthritis, mialgia, or limp are the most common musculoskeletal symptoms. Occasionally, these symptoms are the only ones at the time of presentation, with subtle or absent peripheral blood changes [2, 3].

Articular manifestations can be explained by infiltration of the synovium, periosteum, or bone marrow by malignant cells. They can also be caused by the deposition of uric acid or immune complexes in the joint. A rare cause is hemarthrosis secondary to thrombocytopenia [2–4]. The joint effusion may be secondary to inflammation of the adjacent metaphysis, rather than direct invasion by blasts. Occasionally, as a result of severe infiltration of the metaphysis, the joint appears to be inflamed, simulating juvenile idiopathic arthritis. Severe nocturnal pain that awakens the child, pain that is out of proportion to clinical findings, or pain that does not respond to analgesics, is highly suggestive of malignancy [2, 3]. For this reason, hematological malignancies must be ruled out in patients with musculoskeletal pain, and for this reason, the physician needs to have tools to help discriminate malignant causes from benign ones, improving early diagnosis, treatment, and patient prognosis.

Based solely on musculoskeletal pain, it is difficult to diagnose acute lymphoblastic leukemia, since musculoskeletal manifestations can mimic JIA, osteomyelitis, septic arthritis, or transient synovitis [2]. Clinicians evaluating children with musculoskeletal symptoms should be aware that some hematologic conditions can present with rheumatologic symptoms and signs, and a high index of suspicion is important for a correct diagnosis. The acute onset of pain, the pattern of joint involvement, periarticular rather than joint pain, predominance at night, presence of hepatomegaly, lymph nodes, petechiae, ecchymosis, abnormal blood counts or with subtle changes, poor response to conventional therapy, and lytic lesions on radiographs warrant further investigation of the causes of musculoskeletal pain [1, 3].

Over the years, several reports have been made on the articular manifestations of hematological malignancies and their difference from JIA. Bone pain, arthralgia, limp, myalgia, true arthritis, night pain, pain out of proportion to clinical findings, elevated DHL, presence of peripheral blood blasts, leukocytosis, anemia, thrombocytopenia or low normal platelet count (150,000–250 000), neutropenia, or even normal leukocyte counts have been reported. In this type of patient in whom leukemia presents with musculoskeletal manifestations, a better prognosis with a longer survival has been observed when compared with those who do not present with musculoskeletal symptoms [2, 4, 7–9].

In 2005, Jones conducted a multicenter study in 71 children diagnosed with acute lymphoblastic leukemia and 206 JIA patients to determine the factors that help differentiate between the two diseases. Of the patients with leukemia, 75% (*n* = 53) had no blasts in peripheral blood at the time of evaluation. These patients were compared with those with JIA. The 3 strongest predictors of ALL were leukopenia (< 4000), low normal platelet count (150–250,000), and history of nocturnal pain. The combination of these values showed a sensitivity of 100% and a specificity of 85% for diagnosis of ALL. The number of joints affected in both groups was similar, and uric acid and LDH were not useful as predictors of ALL [10]. In our study, with 2 of 8 clinical variables and 1 of 3 laboratory variables (excluding anemia), it was possible to correctly classify 100% of the patients with leukemia, which makes our study a useful tool for the differential diagnosis of patients with arthritis.

Louvigne in 2020 conducted a study to identify early clinical and laboratory features that help distinguish between JIA and ALL. In a multicenter study, 49 patients with ALL and 98 with JIA were included. Oligoarthritis (43.9%) predominated in the JIA group, followed by polyarthritis (29.6%), arthritis-related enthesitis (18.4%), systemic arthritis (5.1%), and psoriatic arthritis (3.1%). Of the leukemia group, only one patient presented arthritis, the rest presented musculoskeletal pain. Neutrophils, eosinophils, basophils, monocytes, platelets, and hemoglobin were found to be low in patients with ALL. The presence of hepatomegaly, splenomegaly or adenomegaly (*p* = 0.0001, OR 154), non-articular bone pain (*p *= 0.001, OR 13), fever (*p* = 0.01, OR 4.5), neutrophil count less than 2000 (*p* = 0.002, OR 50), platelets < 300,000 (*p* = 0.004, OR14) were found to be significantly associated with the presence of leukemia. This article concludes with a decision tree in which if the patient presents with osteoarticular pain for at least 1 month, a bone marrow aspirate should be performed if accompanied by hepatomegaly, splenomegaly, or adenomegaly. If not accompanied by the latter, if associated with fever or increased inflammatory markers (CRP > 6, ESR > 20), plus non-articular bone pain and/or general symptoms (asthenia, adynamia or weight loss), neutrophils < 2,000 or platelets < 300,000, a bone marrow aspirate should be performed to rule out ALL as the cause of the symptoms [11]. In our study, we found similar results, except that all our patients had arthritis, which makes the differential diagnosis more difficult.

## Conclusions

The studies mentioned above show us that the clinical manifestations and complete blood count of both diseases are different. We only included patients with true arthritis in our study, which makes it different from previous studies in which most patients had musculoskeletal symptoms. This type of presentation of leukemia with true arthritis makes differential diagnosis more difficult for the pediatric rheumatologist, but with the proposed criteria, patients with suspected leukemia can be appropriately selected and bone marrow aspirate can be performed to establish a diagnosis and appropriate treatment. Prospective studies are needed to help validate our results.

## Data Availability

The datasets used during the current study are available from the corresponding author on reasonable request

## Abbreviations

JIA: Juvenile idiopathic arthritis
LA: Leukemic Arthritis
ALL: Acute lymphoblastic leukemia
ILAR: International League of Associations for Rheumatology
FAB: French-American-British classification of acute lymphoblastic leukemia
ESR: Erythrocyte sedimentation rate.
CRP: C reactive protein
VAS: Visual Analogue Scale
